# Social and psychological capital, and organizational performance among smallholders in Henan, China: serial mediating roles of innovation capability and technology adoption

**DOI:** 10.3389/fsoc.2026.1818280

**Published:** 2026-05-11

**Authors:** Ke Li, Kartinah Ayupp, Irma Yazreen Md Yusoff

**Affiliations:** 1 Faculty of Economics and Business, Universiti Malaysia Sarawak, Kota Samarahan, Malaysia; 2 School of Foreign Languages, Zhoukou Normal University, Zhoukou, China

**Keywords:** China, innovation capability, organizational performance, psychological capital, smallholders, social capital, technology adoption

## Abstract

**Introduction:**

This study examines how social capital and psychological capital among smallholder farmers in Henan Province, China, are translated into organizational performance through innovation capability and technology adoption.

**Methods:**

Drawing on the Resource-Based View, Dynamic Capabilities Theory, and Social Cognitive Theory, the study tested a serial mediation model using survey data from 648 smallholder farmers across 18 prefecture-level cities in Henan Province. The data were analyzed using partial least squares structural equation modeling.

**Results:**

Social capital and psychological capital positively predicted innovation capability and technology adoption, while innovation capability further strengthened technology adoption. Technology adoption was positively associated with organizational performance. After the mediating mechanism was incorporated, the direct effects of social capital, psychological capital, and innovation capability on organizational performance were no longer significant. The indirect effects through technology adoption and the sequential indirect effects through innovation capability and technology adoption were supported.

**Discussion:**

The findings suggest that the performance value of intangible resources is largely indirect. Social embeddedness and psychological agency contribute to performance mainly when they are converted into innovation capability and enacted through sustained technology adoption. This study advances an adoption-centered explanation of value creation among resource-constrained smallholder farmers.

## 1 Introduction

Meta-analyses show that adopting innovative agricultural technologies can increase yields and economic benefits (Ogundari and Bolarinwa, 2018). However, technology adoption remains uneven: socioeconomic constraints limit adoption by disadvantaged farmers, and information friction introduces unpredictable implementation risks (Branca and Perelli, 2020; Magruder, 2018). Therefore, technology adoption is less a single, instantaneous choice than a phased learning and implementation process influenced by uncertainty, learning dynamics, and technological attributes (Chavas and Nauges, 2020). Henan, a major grain-producing province, faces frictions to diffusion due to labor out-migration and aging, which reduce farm capacity, while fragmented plots blunt the returns to subsidies and credit (Gao et al., 2023; Zou et al., 2023). Prior research on smallholder adoption has largely foregrounded tangible endowments, especially financial and human capital (Godoy et al., 2000; Wang et al., 2021). Evidence on intangible resources is comparatively fragmented: socio-psychological factors such as social capital and psychological resources shape adoption decisions (Bukchin and Kerret, 2020b; Zeweld et al., 2018), but are seldom integrated into mechanism-based accounts explaining how intangibles translate into sustained adoption and, ultimately, performance.

Social capital (SC) captures farmers' embeddedness in local networks and participatory ties that enable information exchange, reciprocity, and coordination. By facilitating peer-to-peer learning through trusted ties, social capital can reduce search and coordination costs, narrow information asymmetries, and strengthen farmers' capacity to evaluate and manage production risks (Kos et al., 2023). Conceptually, it is embedded in both network structures (e.g., network size and tie strength) and relational qualities (especially trust) that shape access to information and the willingness to coordinate around innovation. However, network resources are most likely to generate sustained behavioral change when coupled with psychological resources that help individuals mobilize social inputs into action; existing evidence suggests social capital can channel psychological capital into innovative behavior (De Carolis and Saparito, 2006; Hidayat et al., 2024).

Psychological capital (PsyCap/PC), comprising hope, self-efficacy, optimism, and resilience, refers to a set of positive, state-like psychological resources that support initiative, persistence, and adaptive coping under uncertainty (Alessandri et al., 2018; Fang et al., 2020; Loi et al., 2025). Meta-analytic evidence indicates that PsyCap is reliably associated with higher performance and desirable attitudes and behaviors, highlighting its relevance as a developable resource for sustained effort and problem-solving in challenging contexts (Avey et al., 2011). For smallholders facing volatile markets and recurrent shocks, PsyCap can facilitate experimentation and sustained adoption by supporting self-control and long-horizon goal pursuit in the face of setbacks (Bukchin and Kerret, 2020a; Zeweld et al., 2019). Yet research that jointly models social capital and PsyCap remains limited, despite indications that peer interactions may nurture psychological resources and that integrated capital perspectives can better explain microenterprise performance than approaches centered on tangible assets alone (Arikan et al., 2025; DelVecchio et al., 2024).

Another limitation in the literature is the lack of a unified mechanism linking intangible resources to downstream outcomes. While correlations between resources and performance are frequently reported, evidence generally suggests that intangible resources create value primarily through intermediate capacity-building processes (e.g., dynamic capabilities or innovation) rather than direct effects (Monteiro et al., 2017; Rua, 2018). Innovation capability (IC) is built through learning-oriented processes (e.g., experimentation, knowledge representation, and encoding), which can act as a bridge, transforming resource potential into viable applications (Lawson and Samson, 2001; Zollo and Winter, 2002). This mechanism is particularly important for resource-poor smallholder farmers in Henan Province. In these regions, funding and technological investment alone are insufficient for the sustained promotion of high-quality technologies; they often only achieve incremental rather than comprehensive adoption (Gao et al., 2019; Jiang et al., 2025).

To address these shortcomings, we integrate the Resource-Based View (RBV), Dynamic Capabilities Theory (DCT), and Social Cognitive Theory (SCT). The Resource-Based View posits that social and psychological capital are strategically significant intangible resources that are both valuable and difficult to replicate, thus possessing performance potential (Barney, 1991). DCT explains how this potential is realized through capability development and deployment, through conscious learning that transforms resources into repeatable and implementable processes, thereby driving performance improvement (Zollo and Winter, 2002; Zott, 2003). SCT complements these perspectives by emphasizing a triadic reciprocal determinism: individual factors, behavior, and environment collectively shape behavioral outcomes (Bandura, 2018). Combining these perspectives, we propose an adoption-centric mechanism in which social and psychological capital strengthen innovation capability, thereby promoting technology adoption among smallholder farmers and improving their organizational performance.

Empirically, we test this mechanism using survey data from 648 smallholder households across 18 prefectures in Henan. Using PLS-SEM, we address two research questions:

RQ1. What structural relationships link social capital and psychological capital with innovation capability and technology adoption among smallholders?

RQ2. What mechanism translates these intangible resources into organizational performance via innovation capability and technology adoption?

This study makes three contributions. First, it develops an adoption-centered integration of the Resource-Based View (RBV), Dynamic Capabilities Theory (DCT), and Social Cognitive Theory (SCT) to explain how intangible resources are translated into capability formation, behavioral implementation, and downstream outcomes in resource-constrained smallholder settings. Second, it clarifies that the value of social capital and psychological capital is likely to be indirect and conditional rather than immediate. Third, it highlights the complementary roles of social embeddedness and psychological agency in shaping innovation capability and technology adoption among smallholders.

## 2 Literature review

Social capital (SC) refers to trust-based networks, reciprocal norms, and shared local cognitions within smallholder communities, which facilitate information access, mutual assistance, and collective coordination (Miao et al., 2015; Sanginga et al., 2007). From a Resource-Based View (RBV) perspective, social capital is a valuable, socially complex, and difficult-to-imitate intangible resource that can reduce search costs, mitigate information asymmetry, and support collective action, thereby creating performance potential (Gold et al., 2009; Tipu and Fantazy, 2018). Dynamic Capabilities Theory (DCT) further clarifies how this potential can be translated into action: by improving the timeliness, credibility, and availability of innovation-related information, connections embedded in social relationships can help farmers perceive opportunities, reduce uncertainty in the trial and implementation process, and more effectively coordinate local responses (Ellonen et al., 2011). Social Cognitive Theory (SCT) complements the above explanation by emphasizing the behavioral roles of trustworthy peers and credible local actors in observational learning, norm reinforcement, and risk reduction. All these factors increase the likelihood of social information being translated into action (Bandura, 2018; Hu et al., 2025; Sander et al., 2024; Savari et al., 2024).

Empirically, SC is expected to enhance innovation capability because trust-based and participatory relationships facilitate cyclical knowledge exchange, peer interaction, and collaborative problem-solving within local agricultural communities. Farmers integrated into trustworthy and information-rich networks are better positioned to acquire, interpret, and integrate practice-related knowledge, thereby reinforcing learning-oriented routines and adaptive experiments (Weber and Heidenreich, 2016; Joffre et al., 2020; Pratiwi and Suzuki, 2017). SC is also expected to promote technology adoption, as peer learning, local demonstration effects, and collective arrangements can reduce uncertainty, increase the credibility of available information, and mobilize input, coordination, and implementation support (Manda et al., 2020; Mumin and Goetz, 2025). In addition, SC may retain a direct association with organizational performance in smallholder settings because informal risk-sharing, labor pooling, collective marketing, and reduced transaction frictions can improve the conditions under which productive activities are organized and sustained (Andersson and Gabrielsson, 2012; Fischer and Qaim, 2014; Markelova and Mwangi, 2010). Such effects are likely to be more contingent and less uniform than the upstream relationships linking SC to innovation capability and technology adoption, but they remain theoretically plausible in contexts where local collective action directly shapes production and market access. We therefore hypothesize:

**H1a:** Social capital is positively associated with innovation capability.

**H1b:** Social capital is positively associated with technology adoption.

**H1c:** Social capital is positively associated with organizational performance.

SC may also influence organizational performance indirectly through capability-building and behavioral implementation. First, SC may enhance performance through innovation capability if trusted ties and local interaction strengthen farmers' capacity to learn, experiment with, and refine improved practices. From a capability perspective, such learning-oriented routines help convert socially embedded resources into more workable and context-adapted ways of operating (Lawson and Samson, 2001; Zollo and Winter, 2002). Second, SC may affect organizational performance through technology adoption when socially embedded support facilitates the sustained implementation of improved practices under conditions of uncertainty, rather than merely encouraging initial acceptance (Chavas and Nauges, 2020; Glover et al., 2019). In addition, a serial pathway is theoretically plausible whereby social capital strengthens innovation capability, innovation capability deepens technology adoption, and technology adoption subsequently contributes to organizational performance. Accordingly, we hypothesize:

**H6a:** Innovation capability mediates the association between social capital and organizational performance.

**H6b:** Technology adoption (TA) mediates the association between social capital and organizational performance.

**H8:** The association between social capital and organizational performance is transmitted sequentially via innovation capability and technology adoption.

Psychological capital (PsyCap) is a developable, state-like positive psychological resource comprising self-efficacy, optimism, resilience, and hope, all of which support initiative-taking, persistence, and adaptive coping in the face of uncertainty (Avey et al., 2011; Dawkins et al., 2013). SCT positions PsyCap as a core source of human agency: self-efficacy strengthens perceived control over challenging tasks, optimism and hope sustain goal-directed effort, and resilience supports recovery when setbacks occur (Avey et al., 2011; Bandura, 2018; Bouckenooghe et al., 2019). From an RBV perspective, PsyCap is a valuable intangible asset because it enhances the effectiveness of other resources by supporting effort, self-regulation, and motivational persistence. From a DCT perspective, PsyCap contributes to reconfiguration by sustaining attention, experimentation, and persistence during trial-and-error correction and routine adjustment under constrained conditions (Luthans et al., 2021).

Empirically, PsyCap is expected to strengthen innovation capability because psychologically resourceful farmers are more proactive in seeking information, experimenting, and learning from feedback, and are better able to recombine knowledge into workable routines (Loi et al., 2025). Psychological capital is also thought to promote technology adoption because optimism and resilience can mitigate fear of failure and perceived downside risk, while self-efficacy can enhance confidence in learning and operating unfamiliar technologies (Diedericks et al., 2019; Fang et al., 2020). Previous research has also shown that positive psychological resources may be directly related to organizational performance by supporting perseverance, disciplined effort, recovery from setbacks, and goal-oriented coping mechanisms in adversity (Avey et al., 2011; Alessandri et al., 2018). In smallholder environments, production conditions are often unstable, and implementation is rarely smooth, so this direct impact is unlikely to be entirely independent of subsequent capacity building and adoption processes; nevertheless, this positive direct association remains theoretically plausible because psychological agency can influence farmers' ability to persevere and cope with adversity in daily production. We therefore hypothesize:

**H2a:** Psychological capital is positively associated with innovation capability.

**H2b:** Psychological capital is positively associated with technology adoption.

**H2c:** Psychological capital is positively associated with organizational performance.

SC and PsyCap are not treated here as competing explanations, but as complementary upstream resources. SC provides socially embedded access to information, credibility, and coordination opportunities, whereas PsyCap supports the confidence, persistence, and adaptive agency required to act on such support under uncertainty. In smallholder settings, access to supportive ties does not by itself guarantee effective enactment, and psychological agency without enabling relational environments may also remain constrained. Their joint inclusion, therefore, allows a more complete explanation of how external embeddedness and internal agency together contribute to capability-building and technology-related change (De Carolis and Saparito, 2006; Hidayat et al., 2024).

Psychological capital may also influence organizational performance indirectly through innovation capability and technology adoption. Farmers with stronger self-efficacy, hope, optimism, and resilience are more likely to persist in learning, experimentation, and adaptive adjustment, thereby strengthening innovation capability as an upstream conversion mechanism (Bandura, 2018; Loi et al., 2025). Psychological capital may also support organizational performance through technology adoption because greater confidence, resilience, and goal-directed persistence can reduce psychological resistance to change and sustain implementation effort under uncertainty (Diedericks et al., 2019; Fang et al., 2020). In addition, a serial pathway is theoretically plausible whereby psychological capital strengthens innovation capability, innovation capability promotes deeper technology adoption, and technology adoption subsequently contributes to organizational performance. Accordingly, we hypothesize:

**H7a:** Innovation capability mediates the association between psychological capital and organizational performance.

**H7b:** Technology adoption mediates the association between psychological capital and organizational performance.

**H9:** The association between psychological capital and organizational performance is transmitted sequentially via innovation capability and technology adoption.

Innovation capability (IC) refers to repeatable, learning-oriented routines involving knowledge search, experimentation, assimilation, recombination, and adaptive adjustment that enable farmers to develop implementable, context-adapted practices (Daronco et al., 2022; Jalotjot and Tokuda, 2024; Makhloufi et al., 2021). From a DCT perspective, IC can be understood as a dynamic capability that operationalizes sensing, seizing, and reconfiguring by translating resource endowments, including SC and PsyCap, into deployable routines for action (Daronco et al., 2022; Teece, 2007). Consistent with RBV, the value of resource advantages depends on whether they are supported by complementary organizational actions and by processes of accumulation, deployment, and renewal (D'Oria et al., 2021; Lavie, 2012). SCT further suggests that capability-building is reinforced through mastery experience, iterative feedback, and adaptive learning during repeated trials, thereby strengthening farmers' capacity to act under uncertainty (Bandura, 2018; Zollo and Winter, 2002).

On this basis, IC is expected to promote technology adoption by enabling farmers to pilot innovations, adapt them to local constraints, and routinize effective practices rather than adopting them only superficially (Brown et al., 2022; Dormon et al., 2007; Taylor and Bhasme, 2024). IC may also retain a direct association with organizational performance because learning-oriented and adaptive capabilities can improve process quality, problem-solving, and the effective adjustment of farming practices under changing production conditions (Arranz et al., 2019; Ravichandran and Lertwongsatien, 2005). At the same time, capability-based research also suggests that a substantial share of IC's performance relevance is likely to be realized when such capability is translated into sustained implementation rather than remaining at the level of potential alone (Lawson and Samson, 2001; Zollo and Winter, 2002). Accordingly, the direct IC–OP relationship remains theoretically plausible, even though technology adoption is expected to serve as the more proximate route through which capability is converted into downstream performance outcomes. We therefore hypothesize:

**H3:** Innovation capability is positively associated with technology adoption.

**H4:** Innovation capability is positively associated with organizational performance.

Technology adoption (TA) is conceptualized in this study as an implementation process involving the appraisal, trial, adaptation, and routinization of improved practices in day-to-day operations rather than as a one-off uptake decision, consistent with research that treats adoption as staged learning and reconfiguration under uncertainty (Chavas and Nauges, 2020; Glover et al., 2019). From an RBV perspective, sustained adoption can transform improved practices into farm-specific production assets and integrate them into the production system, forming stable routines (Haberli Junior et al., 2019). From a DCT perspective, this reflects the process of seizing opportunities and maintaining implementation quality by reconfiguring operational processes as the environment changes (Wang et al., 2025). SCT also points out that adoption and maintenance depend not only on intention but also on repetitive behavior: efficacy beliefs and self-regulation processes support practice, while outcome expectations and supportive cues from peers contribute to long-term sustained use (Cao et al., 2023; Savari et al., 2022). Consistent with this view, the relationship between intention and actual behavior is generally weaker than the influence of efficacy and self-regulation processes, which helps explain why initial intentions may not necessarily translate into sustained implementation (Niles et al., 2016; Schwarzer and Luszczynska, 2008; Sheeran and Webb, 2016).

Empirical studies have shown that technology adoption is generally associated with increased farm profitability, including higher yields, greater household income, and lower production costs (Mulungu et al., 2025; Nguyen et al., 2025). However, these benefits depend on sustained and high-quality implementation; without adequate guidance and follow-up, encouraged adoption may not translate into measurable productivity gains (Kyratsis et al., 2012; Meyers et al., 2012; Whitehead et al., 2020). TA is therefore treated here as the most proximate behavioral channel through which upstream social and psychological resources, together with innovation capability, are translated into organizational performance. We therefore hypothesize:

**H5:** Technology adoption is positively associated with organizational performance.

Organizational performance (OP) in smallholder agriculture is often understood as multidimensional, encompassing not only productivity and economic returns but also the capacity to withstand and respond to shocks in relevant circumstances (Darnhofer, 2014; Pret et al., 2025). From an RBV-DCT perspective, performance heterogeneity stems less from resource possession alone than from the extent to which those resources are organized into capabilities and repeatedly deployed through disciplined implementation processes (Helfat and Maritan, 2023; Helfat and Peteraf, 2003). SCT further suggests that performance variation reflects the interaction of efficacy-based agency, enabling or constraining social environments, and the sustained enactment of focal practices over time (Castro Campos, 2022; Perry and Davenport, 2020). Because repeated behavior typically explains downstream outcomes more directly than intention alone, capability-building that supports continued adoption remains central to explaining organizational performance in resource-constrained smallholder settings (Savari et al., 2022).

**Figure 1:**
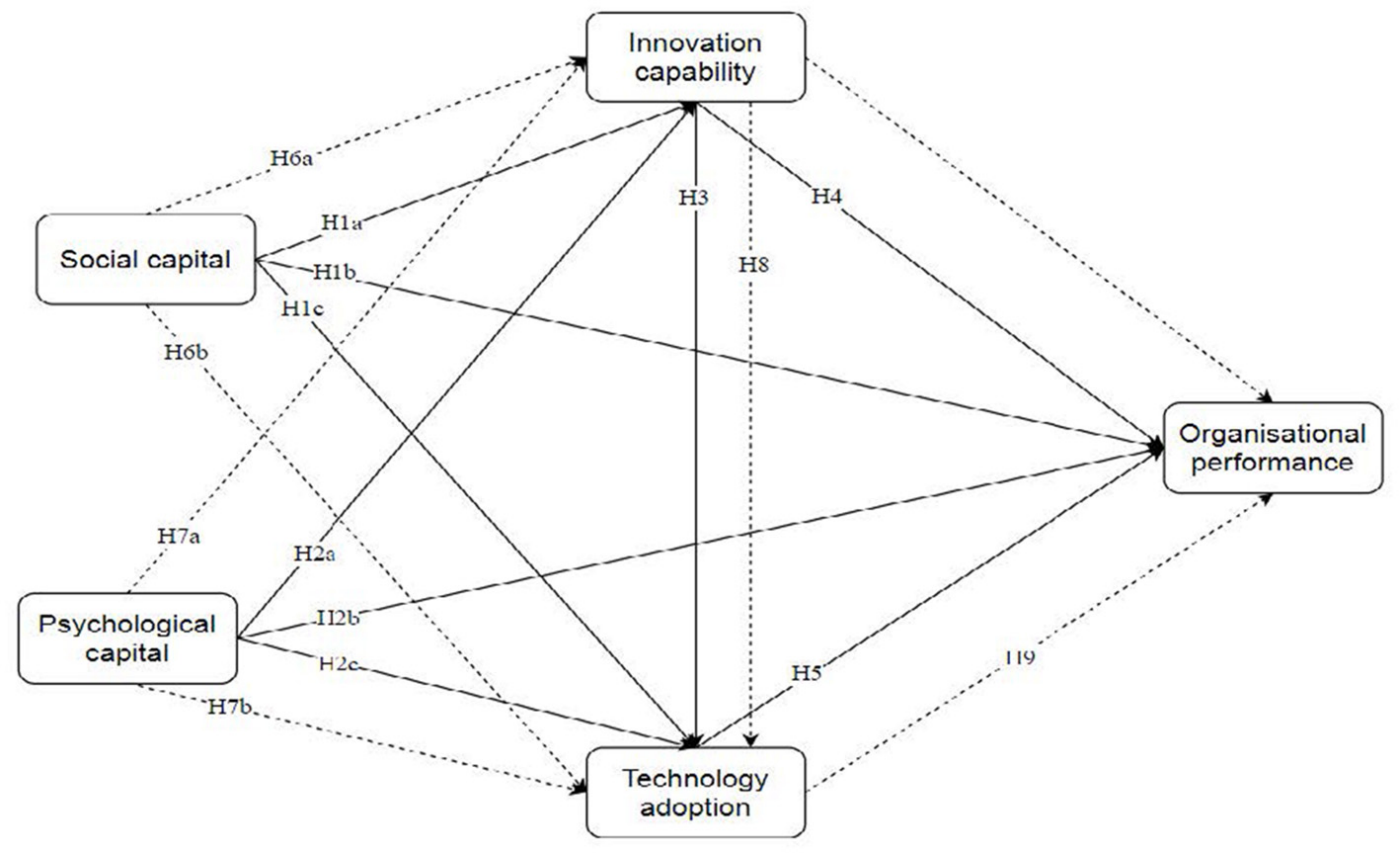
Conceptual framework.

Taken together, these arguments support a conceptual framework in which SC and PsyCap operate as upstream intangible resources, IC functions as a capability-based conversion mechanism, and TA serves as the most proximate behavioral pathway to OP. In this framework, value is expected to arise not simply from resource possession, but from the extent to which intangible resources are converted into workable routines and enacted through sustained implementation. The hypothesized relationships are illustrated in Figure 1.

## 3 Methodology

### 3.1 Research design

This study adopts a cross-sectional survey design to examine whether and how social capital and psychological capital influence organizational performance among smallholder farmers in Henan Province through the mediating roles of innovation capability and technology adoption. In the context of smallholder agriculture, production, resource allocation, and technology-use decisions are typically made at the household level. Accordingly, the family-based farm household is treated as the core decision-making and operating unit. In this study, smallholder farmers are defined as family-based farming households that rely primarily on family labor and operate under relatively small-scale and resource-constrained conditions. Given the absence of a universally accepted threshold that fully captures the diversity of smallholder farming across regions and production systems, this study adopts a context-sensitive definition consistent with the agricultural realities of the study setting.

Henan Province provides an appropriate empirical context for this research. As one of China's major grain-producing regions, it is characterized by the predominance of family-based farming and by continued policy efforts to promote agricultural modernization. At the same time, local agriculture remains constrained by structural challenges such as rural labor outmigration, population aging, and land fragmentation, which may shape farmers' implementation capacity and the practical value of social embeddedness and psychological resources in technology-related decision-making (Gao et al., 2023; Zou et al., 2023). The questionnaire was initially written in English and later translated into Chinese. To ensure semantic equivalence, it was back-translated by professional translators. All discrepancies were carefully reviewed and revised to maintain conceptual accuracy and contextual appropriateness. Prior to the formal survey, the questionnaire was reviewed by local agricultural officials and management scholars, and a pilot test was conducted with 40 smallholder farmers to improve its clarity, sequence, and response flow.

### 3.2 Population, sample and data collection

This study focused on smallholder farmers in Henan Province, China. Data collection employed a stratified multi-stage sampling design, conducted through face-to-face interviews. Eligible respondents were selected based on their recent involvement in agricultural decision-making and active participation in crop cultivation. With the assistance of local village committees, trained investigators followed standardized field procedures throughout the survey process. Initially, 800 questionnaires were collected. After removing invalid responses, 648 valid questionnaires were retained for analysis. This study was approved by the Research Ethics Committee of the Faculty of Economics and Business, Universiti Malaysia Sarawak [UNIMAS; Approval No.: UNIMAS/NC-22.02/09-17 (07)], and informed consent was obtained from all participants prior to data collection.

### 3.3 Measurement items

This questionnaire was developed from existing questionnaires and adjusted to reflect the actual crop cultivation conditions of smallholder farmers in Henan Province. All constructs were measured using a five-point Likert scale, with 1 representing “strongly disagree” and 5 representing “strongly agree”. To improve clarity, Table 1 summarizes the conceptual definitions, sources, number of items, and contextualized operational definitions of the research constructs, and Appendix A lists the measurement items. The model includes five constructs: social capital (SC), psychological capital (PsyCap), innovation capability (IC), technology adoption (TA), and organizational performance (OP). In addition, four control variables were included: education level, farming experience, farm size, and household size. These controls were retained to account for household-level differences that may affect production knowledge, implementation feasibility, labor availability, and resource capacity.

**Table 1:** Conceptual definitions, measurement sources, and contextualized operationalization.

Construct	Conceptual definition	Source	Items	Contextualized operationalization
Social capital (SC)	Social capital refers to resources embedded in local networks and community structures, including participation, trust, reciprocity, and access to support and information channels	Adapted from the World Bank Integrated Questionnaire for the Measurement of Social Capital (SC-IQ) Grootaert, (2004)	4	Reworded to capture village-level farming relations, including participation in village public affairs, generalized trust, mutual help among villagers, and access to farming-related information or assistance through local networks
Psychological capital (PsyCap)	Psychological capital is a developable, state-like positive psychological resource comprising self-efficacy, optimism, resilience, and hope	Adapted from the psychological capital questionnaire (PCQ) Luthans et al., (2007)	4	Items were restated to reflect agricultural and technological applications, covering confidence in mastering new farming techniques, optimism about agriculture, resilience in the face of setbacks, and the ability to find alternative pathways to achieve agricultural goals
Innovation capability (IC)	Innovation capability is the ability to continuously learn, experiment, interpret feedback, and adjust production behavior in response to changing technological and market environments	Adapted from Kump et al. (2019), Saunila and Ukko (2012), and Teece (2007)	4	Operationalized through farmers' ability to improve methods, continuously refine processes, assess whether changes enhance productivity or profitability, and identify and respond to market changes
Technology adoption (TA)	Technology adoption is conceptualized as a phased implementation process reflecting learning, compatibility, sustained use, and routinization in daily operations	Adapted and reworded from UTAUT-related measures Venkatesh et al., (2012)	4	Measured through ease of learning, compatibility with existing farming practices, continued use after initial trial, and integration of new technologies into everyday farming routines
Organizational performance (OP)	Organizational performance refers to the economic outcomes of family-based crop production, with the farming household treated as the relevant decision-making unit	Adapted from subjective comparative performance measures used by White et al. (2020) and Wolff and Pett (2006)	3	Measured through perceived improvement in crop income growth, profitability, and efficiency, with efficiency expressed as higher net profit per unit area during the most recent complete production cycle
Control variables	Variables included to reduce omitted-variable bias and account for structural differences across farming households	Based on prior agricultural household research and model specification logic	4	Education level, farming experience, farm size (cultivated land area), and household size were included as controls in the structural model

SC, social capital; PsyCap, psychological capital; IC, innovation capability; TA, technology adoption; OP, organizational performance. All constructs were measured using items adapted from existing literature and adjusted for context. Detailed measurement items are provided in Appendix A, and standardized outer loadings are reported in Table 4.

## 4 Results

### 4.1 Demographic profile of respondents

Table 2 lists the basic information of the 648 valid respondents included in the analysis. The sample was male (61.3%), with 38.7% female respondents. In terms of age, the 45–54 age group had the highest proportion (40.4%), followed by the 55–64 (30.4%) and 35–44 (21.8%) age groups, indicating that the sample consisted mainly of middle-aged and elderly farmers. Most respondents were married (95.5%). Regarding cooperative membership, 61.1% of respondents reported not being members of farmers' cooperatives, while 38.9% reported being members. The respondents generally had low levels of education. The largest proportion of respondents had lower secondary education (46.9%), followed by those with a primary school education (38.4%), while only 1.4% had a university degree or above. Respondents also had considerable agricultural experience: 41.2% had 21–30 years, and another 23.6% had over 30 years.

**Table 2:** Result of demographic profile of respondents (*n* = 648).

Characteristic	Category	Frequency	%
Gender	Male	397	61.3
	Female	251	38.7
Age (years)	25–34	21	3.2
	35–44	141	21.8
	45–54	262	40.4
	55–64	197	30.4
	65–74	24	3.7
	75 and above	3	0.5
Marital status	Single	5	0.8
	Married	619	95.5
	Divorced and widowed	24	3.7
Cooperative membership	Yes	252	38.9
	No	396	61.1
Education (level)	Non-literate	19	2.9
	Primary education	249	38.4
	Lower secondary education	304	46.9
	Upper secondary education	67	10.3
	College diploma or above	9	1.4
Experience in agricultural work (years)	10 and below	67	10.3
	11–20	161	24.9
	21–30	267	41.2
	30 and above	153	23.6

### 4.2 Descriptive statistics analysis

Table 3 presents descriptive statistics for the main continuous household-level characteristics of the retained sample. The average household size is 5.313 people (standard deviation = 1.417), indicating that most respondents live in medium-sized households. The mean number of years of agricultural production experience was 25.483 (*SD* = 9.366), consistent with the age profile reported in Table 2 and suggesting that many respondents had substantial farming experience. The average cultivated agricultural land area in 2023 was 8.521 mu (*SD* = 5.265), with a range of 2.5–40 mu. These descriptive statistics indicate that the sample largely comprises small-scale, family-operated crop-farming households, consistent with the study's focus on smallholders in Henan.

**Table 3:** Descriptive statistics for continuous household-level characteristics (*n* = 648).

Characteristic	Minimum	Maximum	Mean	*SD*
Family household size	2	11	5.313	1.417
Years of experience in agricultural production	5	45	25.483	9.366
Agricultural land area in 2023 (mu)	2.5	40	8.521	5.265

1 mu is approximately equal to 1/15 ha.

**Source:** Authors' calculations based on survey data.

### 4.3 Analysis of structural equation model using partial least squares

#### 4.3.1 Assessment of the measurement model

Before interpreting the hypothesized relationships, the reflective measurement model was evaluated in terms of indicator reliability, internal consistency reliability, convergent validity, and discriminant validity. Indicator reliability was first assessed through the outer loadings of the measurement items. As shown in Table 4, all loadings ranged from 0.721 to 0.850, exceeding the recommended threshold of 0.70. This indicates that the retained indicators contributed satisfactorily to their respective constructs.

**Table 4:** Result of construct validity and reliability.

Construct	Item	Loading	Cronbach's α	rho_a	CR	AVE
Social capital	SC1	0.748	0.775	0.794	0.854	0.594
	SC2	0.760				
	SC3	0.821				
	SC4	0.750				
Psychological capital	PC1	0.725	0.733	0.736	0.833	0.555
	PC2	0.767				
	PC3	0.721				
	PC4	0.766				
Innovation capability	IC1	0.788	0.817	0.830	0.878	0.643
	IC2	0.828				
	IC3	0.772				
	IC4	0.819				
Technology adoption	TA1	0.728	0.762	0.770	0.848	0.584
	TA2	0.771				
	TA3	0.820				
	TA4	0.733				
Organizational performance	OP1	0.850	0.785	0.789	0.874	0.699
	OP2	0.819				
	OP3	0.838				

Internal consistency reliability was then assessed using Cronbach's alpha, rho_a, and composite reliability (CR). Table 4 shows that Cronbach's alpha values ranged from 0.733 to 0.817, rho_a values ranged from 0.736 to 0.830, and composite reliability values ranged from 0.833 to 0.878. All values are above the acceptable threshold of 0.70, indicating satisfactory internal consistency among the five constructs. AVE values range from 0.555 to 0.699, all exceeding the recommended threshold of 0.50. The results in Table 4 demonstrate that the measurement model possesses sufficient index, internal consistency, and convergent validity.

Discriminant validity was assessed using the Heterotrait-Monotrait Ratio of Correlations (HTMT) and the Fornell–Larcker criterion. As shown in Table 5, all HTMT values ranged from 0.106 to 0.549, well below the conservative threshold. In addition, Table 6 shows that the square root of the AVE for each construct exceeded its correlations with all other constructs. The combined evidence from Tables 5, 6 supports satisfactory discriminant validity. On this basis, the reflective measurement model was considered adequate for subsequent structural model assessment.

**Table 5:** Result of discriminant validity using Heterotrait–Monotrait (HTMT) ratio.

Construct	SC	PC	IC	TA	OP
SC	–	0.399	0.350	0.324	0.127
PC	0.399	–	0.418	0.324	0.106
IC	0.350	0.418	–	0.413	0.202
TA	0.324	0.324	0.413	–	0.549
OP	0.127	0.106	0.202	0.549	–

**Table 6:** Result of discriminant validity using Fornell–Larcker criterion.

Construct	SC	PC	IC	TA	OP
SC	**0.771**				
PC	0.304	**0.745**			
IC	0.293	0.330	**0.802**		
TA	0.257	0.245	0.334	**0.764**	
OP	0.097	0.070	0.162	0.427	**0.836**

Bold values on the diagonal represent the square roots of AVE for each construct.

#### 4.3.2 Assessment of the structural model

After establishing the adequacy of the measurement model, the structural model was assessed for collinearity, common-method bias diagnostics, explanatory power, model fit, predictive relevance, and effect sizes. First, collinearity among predictor constructs was examined using the inner Variance Inflation Factors (VIF). As shown in Table 7, all inner VIF values ranged from 1.102 to 1.251, which is well below commonly applied threshold levels. This suggests that multicollinearity was not a concern in estimating the structural relationships.

**Table 7:** Result of common method bias diagnostics using VIFs.

Structural path	VIF
SC → IC	1.102
SC → OP	1.180
SC → TA	1.155
PC → IC	1.102
PC → OP	1.200
PC → TA	1.184
IC → OP	1.251
IC → TA	1.176
TA → OP	1.178

Second, common method bias was assessed as a supplementary diagnostic. Harman's single-factor test showed that the first unrotated factor accounted for 24.99% of the total variance, which is below conventional levels of concern. In addition, the inner VIF values reported in Table 7 were all well below commonly used threshold levels, indicating that problematic multicollinearity was unlikely in the structural model. Third, the explanatory power of the model was assessed using the coefficient of determination (*R*^2^). As shown in Table 8, the model explained 15.0, 15.1, and 18.5% of the variance in IC, TA, and OP, respectively. The corresponding adjusted *R*^2^ values were 0.147, 0.147, and 0.180. These values indicate that the model has limited-to-moderate explanatory power, which is not uncommon in heterogeneous smallholder environments based on field research, where behavioral and performance outcomes are influenced by a variety of contextual factors.

**Table 8:** Explanatory power of the endogenous constructs.

Endogenous construct	*R* ^2^	Adjusted *R*^2^
IC	0.150	0.147
TA	0.151	0.147
OP	0.185	0.180

SRMR was used to assess the model fit descriptively. As shown in Table 9, the SRMR values for both the saturated and estimated models were 0.066, which is lower than the commonly used benchmark of 0.08. These values indicate that the model fit is acceptable.

**Table 9:** SRMR values for the structural model.

Model	SRMR
Saturated model	0.066
Estimated model	0.066

Predictive relevance was further evaluated using PLSpredict. As shown in Table 10, the *Q*^2^_predict values for IC, TA, and OP are 0.139, 0.089, and 0.004, respectively. Since all values are greater than zero, the model has predictive relevance at the construct level. However, the predictive relevance for OP is significantly weaker compared to IC and TA. This suggests that the model is more effective at predicting upstream capability formation and behavior implementation than at predicting downstream performance outcomes, which may be more susceptible to external environmental factors.

**Table 10:** Result of PLSpredict of out-of-sample predictive relevance.

Endogenous construct	*Q*^2^_predict	RMSE	MAE
IC	0.139	0.932	0.712
TA	0.089	0.959	0.729
OP	0.004	1.002	0.794

The effect size, as shown in Table 11, further clarifies the relative contribution of the hypothesized structural paths. Technology adoption showed the largest practical effect on organizational performance (*f*^2^ = 0.192). Among the upstream relationships, psychological capital had a small but meaningful effect on innovation capability (*f*^2^ = 0.075), innovation capability had a small but meaningful effect on technology adoption (*f*^2^ = 0.064), social capital had a small effect on innovation capability (*f*^2^ = 0.048), and social capital had a small effect on technology adoption (*f*^2^ = 0.022). By contrast, the direct effects of social capital, psychological capital, and innovation capability on organizational performance were negligible. Thus, the structural model assessment suggests that the model's explanatory power lies primarily in the resource-capability-adoption sequence rather than in direct resource-to-performance relationships.

**Table 11:** Effect-size estimates (*f*^2^) for the structural paths.

Path	*f* ^2^	*SD*	*t*-value	*p*-value
SC → IC	0.048	0.021	2.336	0.020
SC → TA	0.022	0.011	2.008	0.045
SC → OP	0.000	0.003	0.038	0.970
PC → IC	0.075	0.025	2.990	0.003
PC → TA	0.014	0.010	1.301	0.193
PC → OP	0.002	0.004	0.455	0.649
IC → TA	0.064	0.021	2.960	0.003
IC → OP	0.001	0.004	0.356	0.722
TA → OP	0.192	0.047	4.099	0.001

*f*^2^ values of 0.02, 0.15, and 0.35 are commonly interpreted as small, medium, and large effects, respectively. *SD* = bootstrap standard deviation; *p*-values are reported.

#### 4.3.3 Endogeneity considerations

As an additional robustness check, a Gaussian copula analysis was conducted in SmartPLS to assess whether the structural estimates were materially affected by endogeneity. Before applying the Gaussian copula procedure, the retained indicators were examined for non-normality. The results indicated systematic departures from normality, thereby supporting the use of the Gaussian copula approach as an auxiliary diagnostic specification.

As shown in Table 12, the reported Gaussian copula terms were largely non-significant. The copula terms associated with SC → IC, PC → IC, SC → TA, PC → TA, SC → OP, IC → OP, and TA → OP were not statistically significant. Only the copula term associated with IC → TA approached marginal significance at the 10% level (β = −0.298, *t* = 1.908, *p* = 0.056). Taken together, these results do not indicate strong and systematic endogeneity across the reported structural relationships.

**Table 12:** Gaussian copula terms for endogeneity diagnostics.

Structural relationship tested	β	*SD*	*t*-value	*p*-value	Interpretation
GC (SC → IC) → IC	−0.171	0.482	0.355	0.723	No evidence of endogeneity
GC (PC → IC) → IC	−0.171	0.208	0.823	0.410	No evidence of endogeneity
GC (SC → TA) → TA	−0.055	0.402	0.136	0.892	No evidence of endogeneity
GC (PC → TA) → TA	0.287	0.196	1.464	0.143	No evidence of endogeneity
GC (IC → TA) → TA	−0.298	0.156	1.908	0.056	Marginal at the 10% level
GC (SC → OP) → OP	−0.210	0.330	0.635	0.525	No evidence of endogeneity
GC (IC → OP) → OP	0.107	0.188	0.570	0.569	No evidence of endogeneity
GC (TA → OP) → OP	0.231	0.333	0.693	0.488	No evidence of endogeneity

GC, Gaussian copula term.

Table 13 reports the Gaussian copula-adjusted structural path estimates. After inclusion of the control-function terms, only two substantive paths remained statistically significant: PC → IC (β = 0.433, *t* = 2.066, *p* = 0.039) and IC → TA (β = 0.561, *t* = 3.375, *p* = 0.001). The remaining adjusted paths were not statistically significant. This pattern suggests that the most robust part of the explanatory sequence lies in the pathway from psychological capital to innovation capability and from innovation capability to technology adoption.

**Table 13:** Gaussian copula-adjusted structural path estimates.

Hypothesized relationship	β	*SD*	*t*-value	*p*-value	Result
SC → IC	0.390	0.496	0.785	0.432	Not significant
PC → IC	0.433	0.209	2.066	0.039	Significant
SC → TA	0.205	0.411	0.498	0.618	Not significant
PC → TA	−0.164	0.201	0.815	0.415	Not significant
IC → TA	0.561	0.166	3.375	0.001	Significant
SC → OP	0.291	0.356	0.816	0.414	Not significant
PC → OP	−0.037	0.146	0.253	0.800	Not significant
IC → OP	−0.070	0.178	0.396	0.692	Not significant
TA → OP	0.159	0.348	0.458	0.647	Not significant

The reported coefficients are obtained from the Gaussian copula-adjusted structural model.

Table 14 summarizes the diagnostic results of the model adjusted with the Gaussian Copula. The adjusted model retains moderate explanatory power, with *R*^2^ values of 0.151, 0.158, and 0.188 for IC, TA, and OP, respectively. The SRMR value of the saturated model is 0.066, and the SRMR value of the estimated model is 0.105. Thus, the Gaussian-copula results do not reverse the main directional pattern of the model, but they do suggest that the performance-stage estimates are less stable than the upstream resource-capability-adoption relationships.

**Table 14:** Gaussian copula-adjusted specification.

Diagnostic	Value
*R*^2^ (innovation capability)	0.151
*R*^2^ (technology adoption)	0.158
*R*^2^ (organizational performance)	0.188
SRMR (saturated model)	0.066
SRMR (estimated model)	0.105
d_ULS (saturated model)	0.822
d_ULS (estimated model)	2.093
d_G (saturated model)	0.224
d_G (estimated model)	0.404

### 4.4 Hypothesis testing

#### 4.4.1 Direct effects

The direct effects provide broad support for the proposed upstream mechanism linking intangible resources to capability formation and technology adoption. Social capital positively predicts innovation capability (β = 0.212, *p* < 0.001) and technology adoption (β = 0.148, *p* < 0.001), thereby supporting H1a and H1b. Psychological capital likewise positively predicts innovation capability (β = 0.265, *p* < 0.001) and technology adoption (β = 0.117, *p* = 0.005), thereby supporting H2a and H2b. Taken together, these findings indicate that both external social embeddedness and internal psychological agency contribute to the development of learning-oriented capabilities and the implementation of improved practices.

Innovation capability also positively predicts technology adoption (β = 0.252, *p* < 0.001), supporting H3. This finding reinforces the role of innovation capability as an upstream conversion mechanism through which intangible resources are translated into behavioral implementation. Technology adoption, in turn, is positively associated with organizational performance (β = 0.429, *p* < 0.001), supporting H5. This result is consistent with the theoretical argument developed in Section 2 that technology adoption is the most proximate behavioral route through which upstream resources and capability are translated into downstream performance outcomes.

By contrast, once innovation capability and technology adoption are incorporated into the model, the direct effects of social capital (β = −0.010, *p* = 0.808), psychological capital (β = −0.044, *p* = 0.284), and innovation capability (β = 0.036, *p* = 0.374) on organizational performance are not statistically significant. Accordingly, H1c, H2c, and H4 are not supported. These results suggest that the performance relevance of upstream resources and capability is not immediate or independently realized once the mediating mechanisms are taken into account, but depends primarily on whether they are translated into sustained technology adoption.

#### 4.4.2 Indirect and serial mediation effects

The mediation results further clarify how upstream intangible resources are translated into organizational performance. For both social capital and psychological capital, the indirect effects through technology adoption are statistically significant. Specifically, social capital has a significant indirect impact on organizational performance through technology adoption (β = 0.063, *p* < 0.001), supporting hypothesis H6b; psychological capital also has a significant indirect impact through technology adoption (β = 0.050, *p* = 0.008), supporting hypothesis H7b. These findings suggest that technology adoption is a key behavioral transmission path connecting upstream resources and downstream outcomes.

In contrast, the indirect impact through innovation capability alone is not significant. The path of social capital influencing organizational performance through innovation capability is not supported (β = 0.008, *p* = 0.405), and the corresponding path of psychological capital is also not supported (β = 0.010, *p* = 0.384). Therefore, hypotheses H6a and H7a are not supported. This pattern suggests that the importance of innovation capability lies primarily in its role in facilitating subsequent implementation, rather than its ability to generate measurable performance improvements.

The results of the sequential mediation analysis reinforce this explanation. Social capital has a significant indirect impact on organizational performance through innovation capability and technology adoption (β = 0.023, *p* < 0.001), supporting hypothesis H8. Psychological capital also has a significant sequential indirect impact on organizational performance through innovation capability, which in turn affects technology adoption (β = 0.029, *p* < 0.001), supporting Hypothesis H9. In summary, these findings are consistent with the proposed resource-capability-adoption-performance sequence: intangible resources enhance innovation capability, innovation capability deepens technology adoption, and performance benefits are primarily realized when improvement practices are implemented and maintained.

**Figure 2:**
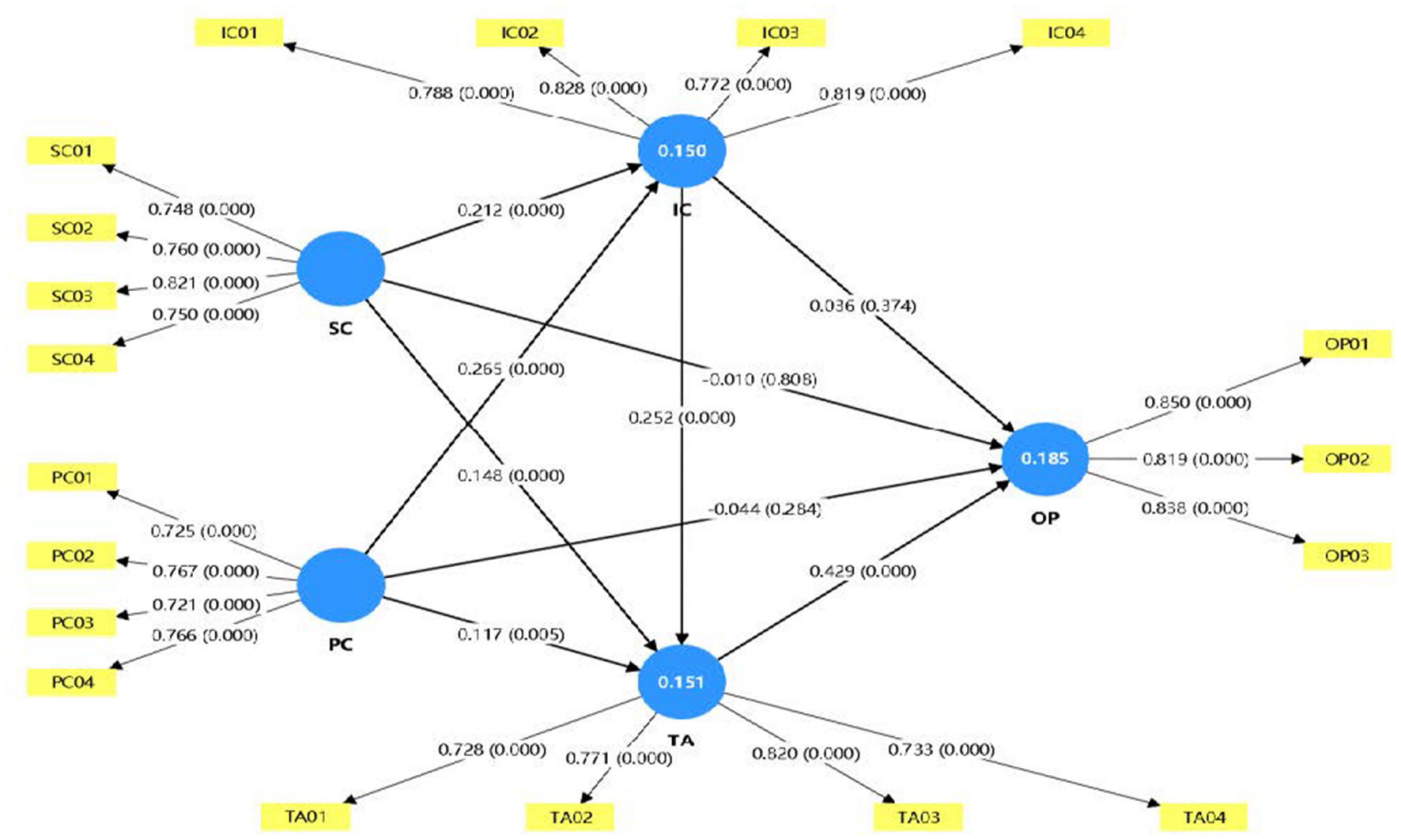
PLS-SEM structural model.

Figure 2 displays the estimated direct structural paths in the final PLS-SEM model, whereas Table 15 reports the bootstrapped direct, indirect, and serial effects used to evaluate the hypotheses.

**Table 15:** Results of direct, indirect, and serial effects.

Hypothesis	Relationship	β	*SD*	*t*-value	*p*-value	2.5%	97.5%	Result
Direct effects								
H1a	SC → IC	0.212	0.042	5.017	0.001	0.131	0.295	Supported
H1b	SC → TA	0.148	0.035	4.264	0.001	0.080	0.217	Supported
H1c	SC → OP	−0.010	0.043	0.243	0.808	−0.095	0.072	Not supported
H2a	PC → IC	0.265	0.040	6.582	0.001	0.188	0.347	Supported
H2b	PC → TA	0.117	0.042	2.788	0.005	0.033	0.200	Supported
H2c	PC → OP	−0.044	0.041	1.071	0.284	−0.124	0.038	Not supported
H3	IC → TA	0.252	0.039	6.479	0.001	0.176	0.327	Supported
H4	IC → OP	0.036	0.041	0.889	0.374	−0.046	0.116	Not supported
H5	TA → OP	0.429	0.043	9.977	0.001	0.346	0.512	Supported
Indirect effects								
H6a	SC → IC → OP	0.008	0.009	0.832	0.405	−0.009	0.027	Not supported
H6b	SC → TA → OP	0.063	0.016	3.914	0.001	0.034	0.097	Supported
H7a	PC → IC → OP	0.010	0.011	0.871	0.384	−0.012	0.032	Not supported
H7b	PC → TA → OP	0.050	0.019	2.649	0.008	0.014	0.089	Supported
Serial mediating effect								
H8	SC → IC → TA → OP	0.023	0.007	3.524	0.001	0.012	0.037	Supported
H9	PC → IC → TA → OP	0.029	0.007	4.119	0.001	0.017	0.044	Supported

β = standardized path coefficient; *SD* = bootstrap standard deviation; 2.5 and 97.5% = lower and upper bounds of the bootstrap 95% confidence interval. *p*-values are reported.

## 5 Discussion and implications

### 5.1 Discussion

This study examined how social capital and psychological capital are translated into smallholder organizational performance through innovation capability and technology adoption. Taken together, the findings support an adoption-centered interpretation of value creation in which intangible resources create performance potential, but generate reliable benefits primarily when they are converted into learning-oriented capability and subsequently enacted through sustained technology adoption. The structural model provides the primary basis for inference, while the Gaussian copula analysis serves as an auxiliary robustness check. The results should therefore be interpreted not as support for a simple direct resource-to-performance model, but as evidence of a conditional process in which capability formation and behavioral implementation play the central mediating role.

First, the positive associations of social capital and psychological capital with innovation capability and technology adoption indicate that intangible resources matter through both social embeddedness and individual agency. Social capital reduces information friction, enhances the credibility of existing knowledge, and facilitates coordination in the trial and implementation process. In smallholder communities, trust-based and reciprocal relationships help farmers more easily access available information, observe local cases, and obtain practical support during adoption (Kos et al., 2023; Savari et al., 2024; Zheng, 2010). Psychological capital operates differently but is closely related to social capital. It supports farmers' willingness and ability to cope with uncertainty by enhancing self-efficacy, optimism, resilience, and goal-oriented persistence, enabling them to continue learning after setbacks and persevere when encountering difficulties and imperfections in practice (Avey et al., 2011; Bouckenooghe et al., 2019; Dawkins et al., 2013). In summary, these results support the view that technological change among smallholders depends not only on access to favorable social environments but also on their intrinsic psychological capacity to utilize such support. In this sense, these findings align with the “co-empowerment” perspective, which posits that social embeddedness and psychological agency are complementary rather than competitive foundations for the formation and adoption of capability (Bandura, 2018; De Carolis and Saparito, 2006; Hidayat et al., 2024).

Secondly, innovation capability can positively predict technology adoption, but once technology adoption is considered, it no longer has an independent direct impact on organizational performance. This model clarifies the role of innovation capability as an upstream transformation mechanism, rather than a direct performance driver. From a DCT perspective, the value of resources depends not only on their availability but also on whether these resources are organized into deployable processes that can be implemented, improved, and maintained over time (Helfat and Peteraf, 2003; Zollo and Winter, 2002). In this study, innovation capability encompasses a range of routine processes, including search, experimentation, evaluation, reorganization, and adaptive adjustment. These processes enable farmers to pilot improved farming methods, interpret feedback, and adjust implementation plans to deepen subsequent adoption (Brown et al., 2022; Lawson and Samson, 2001; Taylor and Bhasme, 2024). Therefore, the seemingly weak direct path between innovation capability and organizational performance is theoretically significant, not merely ineffective. This suggests that capacity does not automatically generate measurable rewards; rather, its value is realized only when it is translated into repetitive behaviors and consistently applied in daily farming practices.

Third, technology adoption is the most direct way in which intangible resources and innovation capability are transformed into organizational performance. Once innovation capability and technology adoption are incorporated into the model, the direct impact of social capital, psychological capital, and innovation capability on performance becomes negligible, while the indirect impact through technology adoption and the sequential path of innovation capability preceding technology adoption remain valid. This model aligns with resource-based theory, which posits that resources create performance potential, but the actual advantage gained depends on how these resources are mobilized, combined, and deployed in practice, not merely on resource ownership (Barney, 1991; Helfat and Maritan, 2023; Lavie, 2012). It is also consistent with adoption-based perspectives, which view adoption not as a one-off decision but as a phased process of evaluation, trial, adaptation, and routinization under uncertainty (Chavas and Nauges, 2020; Glover et al., 2019). Mediation effects reinforce this explanation. Technology adoption significantly transmits the influence of social and psychological capital to organizational performance, and the cascading path between innovation capability and technology adoption is also supported. In conclusion, the findings suggest that the performance value of intangible resources is indirect, conditional, and implementation-dependent, rather than immediate.

Finally, the explanatory and predictive results indicate that the boundary conditions of the performance phase are clearer than those of the upstream capability and adoption phases. While the model demonstrates significant explanatory power overall, predictive assessments show that its out-of-sample relevance is stronger in terms of innovation capability and technology adoption than in terms of organizational performance. This asymmetry is theoretically plausible in smallholder agriculture, where the learning and implementation processes are more closely linked to core mechanisms, while downstream performance outcomes are influenced by a wider range of contingent factors outside the model. These may include market access, input quality, weather variability, implementation support, and other household- or context-specific shocks that affect whether adoption is ultimately converted into measurable performance gains (Darnhofer, 2014; Pret et al., 2025; Quinlan et al., 2016). The robustness analysis is consistent with this interpretation, as it suggests that the resource-capability-adoption sequence is more stable than the final adoption-to-performance conversion. Accordingly, the present findings support a mechanism-based explanation of how intangible resources are translated into capability and adoption, while also indicating that organizational performance remains a more conditional and context-sensitive outcome.

### 5.2 Practical implications

Given that social capital and psychological capital are linked to organizational performance primarily through technology adoption, practical interventions should focus less on treating these resources as ends in themselves and more on improving the mechanisms through which they are converted into effective implementation. A first implication is the need to institutionalize trusted peer-learning structures that strengthen adoption routines rather than relying solely on one-way information delivery. The positive role of social capital suggests that farmers are more likely to adopt and sustain improved practices when innovations are embedded in repeated, credible, and locally meaningful social interaction. Trust relations can reduce ambiguity, increase the credibility of information, and support observational learning during trial and early implementation (De Wever et al., 2005; Savari et al., 2024; Zheng, 2010). At the practical level, extension systems and local agricultural organizations should place less emphasis on one-off or fragmented training and give greater attention to structured peer-learning groups, local demonstration networks, and pilot platforms that incorporate feedback. These arrangements allow farmers to observe, discuss, test, and gradually incorporate new practices into their everyday farming activities. They are also more consistent with the mechanism identified in this study, as they help turn informal social resources into more organized forms of learning and implementation support (Joffre et al., 2020; Manda et al., 2020).

A second implication is that psychological capital should be regarded not simply as an individual trait difference, but as something that can be strengthened through appropriate support. Its positive association with innovation capability and technology adoption suggests that farmers with greater self-efficacy, optimism, resilience, and hope are better placed to experiment, persist, and adjust when facing change. This is important because implementation is often difficult in practice. Trial-and-error learning, early setbacks, and uncertainty about outcomes can easily discourage continued use unless farmers have enough confidence and resilience to maintain their effort over time (Bandura, 2018; Schwarzer and Luszczynska, 2008; Sheeran and Webb, 2016). For this reason, support programmes should include phased trials, timely feedback, peer encouragement, and practical troubleshooting so that farmers can gain early confidence, recover from setbacks, and continue using new practices. Such arrangements are more likely to support sustained adoption than the simple provision of technical information alone (Avey et al., 2011; Bouckenooghe et al., 2019).

A third implication is that soft enabling conditions need to be accompanied by practical implementation support if post-adoption difficulties are to be reduced. The findings suggest that, although social capital and psychological capital matter, they are not sufficient on their own to generate performance gains unless the conditions for feasible and sustained implementation are also present. In smallholder farming contexts, even when farmers are willing to try and able to learn, adoption may still fail to produce meaningful outcomes because of resource constraints, limited access to inputs, technical uncertainty, or weak follow-up support (Branca and Perelli, 2020; Magruder, 2018). Support strategies should therefore connect social learning and agency-building with the material and organizational conditions needed for continued implementation. More effective programmes are likely to combine peer learning, confidence-building, and local coordination with trial inputs, follow-up visits, troubleshooting support, and implementation guidance that extends beyond initial uptake (Kyratsis et al., 2012; Meyers et al., 2012; Whitehead et al., 2020). This bundled approach is more consistent with the present findings because it directly supports the shift from resource potential and capability to sustained use in operation.

A fourth implication is that monitoring systems should follow the full resource-capability-adoption-performance sequence rather than focusing only on adoption rates. The predictive results indicate that downstream performance is more difficult to capture and predict than upstream capability and adoption processes. This suggests that monitoring should not be limited to whether a practice has been adopted, but should also assess the depth of integration, consistency of implementation, and the conditions under which adoption translates into measurable outcomes. This is consistent with viewing adoption as a staged process of learning, adaptation, and routinization rather than as a one-off event (Chavas and Nauges, 2020; Glover et al., 2019). In practice, a more useful monitoring architecture would include at least three layers: first, indicators of social and psychological resource formation, such as participation, trust, self-efficacy, and persistence; second, indicators of innovation capability and adoption depth, such as experimentation frequency, routinization, and the breadth of practice integration; and third, indicators of performance-conversion conditions, combining subjective outcome assessments with feasible objective proxies where possible. In smallholder contexts, subjective performance measures are often unavoidable and can still provide useful information. At the same time, combining them with other sources of evidence can strengthen confidence in the interpretation of the results, both by reducing common-source bias and by clarifying whether adoption has led to better outcomes (Pret et al., 2025; White et al., 2020; Wolff and Pett, 2006). This would help practitioners judge more accurately where the conversion process is working, where it begins to break down, and what kind of support is needed at different points.

### 5.3 Theoretical contributions

First, this study develops a more integrated mechanism-based explanation by organizing the Resource-Based View (RBV), Dynamic Capabilities Theory (DCT), and Social Cognitive Theory (SCT) into a coherent sequence of resource mobilization, capability formation, behavioral implementation, and downstream performance. Rather than treating these perspectives as parallel background lenses, the study assigns them distinct explanatory roles within a single process model. RBV provides the resource logic by framing social capital and psychological capital as upstream intangible assets with performance potential (Barney, 1991; Lavie, 2012). SCT contributes to the behavioral micro-foundation by explaining why social embeddedness and psychological agency should influence farmers' willingness and capacity to learn, experiment, and act under uncertainty (Bandura, 2018). DCT clarifies why such resource potential is not self-executing but must be converted into deployable routines and then sustained through capability-building and reconfiguration (Helfat and Peteraf, 2003; Zollo and Winter, 2002). The contribution, therefore, lies not merely in combining three theories, but in showing how they can be organized into a single explanatory sequence linking upstream intangibles to downstream outcomes in a resource-constrained smallholder context.

Second, the study refines the literature on intangible resources and performance by showing that the value of social capital and psychological capital is indirect, conditional, and implementation-dependent rather than immediate. Once innovation capability and technology adoption are incorporated into the model, the direct effects of social capital, psychological capital, and innovation capability on organizational performance are no longer significant, whereas the indirect effects through technology adoption and the serial paths through innovation capability followed by technology adoption remain supported. In particular, the findings distinguish between two different types of mediation logic. Mediation through innovation capability alone would imply that capability-building can translate resource advantages into performance more directly, whereas the supported serial pattern suggests that resource advantages are more likely to yield downstream benefits when they are first converted into learning-oriented capability and then into sustained behavioral implementation. In this respect, the study contributes to RBV- and DCT-informed research by clarifying that the performance value of intangible resources lies less in their possession than in their enactment through routinized adoption under conditions of implementation friction (Chavas and Nauges, 2020; Glover et al., 2019; Lawson and Samson, 2001; Zollo and Winter, 2002).

Third, the study contributes to smallholder innovation research by demonstrating the complementarity of social embeddedness and psychological agency. Social capital and psychological capital should not be treated as competing explanations for adoption-related outcomes, but as distinct yet mutually reinforcing enablers of capability-building and implementation. Social capital provides trusted ties, locally credible information, and coordination opportunities that reduce information frictions and strengthen peer-based learning. Psychological capital contributes through a different but complementary route by supporting confidence, persistence, adaptive coping, and goal-directed effort when experimentation and implementation are difficult or uncertain (Avey et al., 2011; Bouckenooghe et al., 2019). The joint modeling of these two forms of intangible capital, therefore, extends existing work that tends to privilege either external relational resources or internal psychological resources in isolation. More importantly, the findings suggest that sustained technology-related change in smallholder settings depends not only on whether farmers can access support and information through their social environment, but also on whether they possess the psychological capacity to act on such inputs, persist through setbacks, and stabilize repeated use in practice. In this respect, the study deepens SCT-informed reasoning in agriculture by showing that person, environment, and behavior are linked through a concrete sequence of capability formation and adoption enactment rather than through broad conceptual association alone.

## 6 Conclusion, limitations and future research

This study examined how social capital and psychological capital are translated into smallholder organizational performance through innovation capability and technology adoption. The findings support an adoption-centered interpretation of value creation, showing that intangible resources generate performance potential but produce consistent benefits primarily when they are converted into learning-oriented capabilities and enacted through sustained technology adoption. Rather than supporting a direct resource-to-performance relationship, the results demonstrate a conditional process in which capability formation and behavioral implementation play central mediating roles. Technology adoption emerges as the most immediate pathway through which intangible resources and innovation capability influence organizational performance.

Once the mediating mechanisms are considered, the direct effects of social capital, psychological capital, and innovation capability on performance become negligible, while the indirect and sequential pathways remain significant. This indicates that the value of intangible resources is largely indirect, depending on their translation into actionable practices rather than their mere possession. Thus, the study advances a mechanism-based explanation of smallholder performance by linking resource mobilization, capability development, and implementation within a single framework. At the same time, the findings highlight that performance outcomes remain context-sensitive and contingent on conditions beyond adoption itself. These insights underscore the importance of focusing not only on resource endowments, but also on the processes that enable their effective conversion into sustained organizational outcomes.

In addition, this study should be interpreted in light of several limitations that also point to directions for future research. First, although the cross-sectional design is appropriate for examining the plausibility of the proposed relationships and mechanisms, it does not support strong causal claims regarding temporal ordering. The endogeneity checks provide useful additional evidence on the robustness of the findings, but they cannot replace research designs that follow how intangible resources, innovation capability, technology adoption, and organizational performance develop over time. Future research could address this limitation by using multi-wave, seasonal, or panel data to examine whether the relationships observed here remain stable across different stages of agricultural production and under different implementation conditions.

Second, technology adoption and organizational performance were measured mainly through self-reports. This is common, and often unavoidable, in smallholder research, where detailed behavioral records and audited financial data are rarely available in a standardized form. Even so, self-reported measures may increase common-source dependence and may not fully reflect actual implementation behavior or changes in performance. Future studies could improve measurement by triangulating survey data with behavioral indicators, administrative records, observational evidence, or selected objective performance measures where feasible.

Third, organizational performance is likely to depend on situational conditions beyond the focal mechanism examined here. Although the framework helps explain how social capital and psychological capital are translated into innovation capability and technology adoption, the conversion from adoption into measurable performance is likely to remain contingent on contextual factors such as market access, input quality, weather variability, local implementation support, and household-level shocks. Future research could extend the model by incorporating these boundary conditions more explicitly, thereby clarifying when adoption is most likely to lead to improved outcomes.

Finally, mixed-methods and comparative research designs could add interpretive depth to the present findings. While structural modeling is useful for identifying systematic relationships among social capital, psychological capital, innovation capability, technology adoption, and organizational performance, it is less able to show how these processes operate at the household and community levels in practice. Future research could combine survey-based modeling with interviews, case studies, or field observations to explore how trust, reciprocity, information exchange, and psychological agency influence adoption trajectories under different conditions. This would help explain why farming households with similar resource endowments may still follow different implementation paths and achieve different performance outcomes.

## Data Availability

The raw data supporting the conclusions of this article will be made available by the authors, without undue reservation.

## Appendix A

**Table A1:** Questionnaire.

Construct	Codes	Item	Source
Social capital	SC1	I actively participate in village public affairs (e.g., attending village meetings, voting, and community decision-making)	Adapted from SC-IQ; Grootaert (2004)
	SC2	In general, most people in this village are trustworthy	
	SC3	People in this village are willing to help each other when needed	
	SC4	When I need farming-related information or assistance, I can usually obtain it through village networks (e.g., village leaders, neighbors, or local groups)	
Psychological capital	PC1	I am confident that I can master new farming techniques when needed	Adapted from PCQ tradition; Luthans et al. (2007)
	PC2	I am optimistic about my farming future and the prospects of agriculture in my area	
	PC3	When I encounter farming setbacks (e.g., market shocks or crop losses), I can recover and continue effectively	
	PC4	Even when farming goals are difficult, I can find alternative ways to achieve them and keep working toward long-term gains	
Innovation capability	IC1	I actively explore and test new cultivation methods and farming practices	Adapted from Kump et al. (2019); Saunila and Ukko (2012); Teece (2007)
	IC2	Over the past few years, I have continuously improved my farming processes (e.g., planning, input use, and field management)	
	IC3	I can evaluate whether improved management practices are likely to increase productivity or profits on my farm	
	IC4	I can detect changes in market demand or conditions and adjust my planting/management decisions accordingly	
Technology adoption	TA1	I can learn to operate new agricultural technologies without much difficulty	Adapted from Venkatesh et al. (2012)
	TA2	I can integrate new agricultural technologies into my existing farming processes without major disruption	
	TA3	After trying new agricultural technology, I usually continue using it rather than abandoning it	
	TA4	Using new agricultural technologies has become part of my routine farming operations	
Organizational performance	OP1	In 2023, my total crop income growth was higher than that of most households in my village	Adapted from White et al. (2020); Wolff and Pett (2006)
	OP2	In 2023, my net profit margin from crop production increased compared with 2022	
	OP3	In 2023, the net profit per unit area of my core crops improved compared with 2022	
